# Pulse wave velocity measurement in the aorta: comparison of pediatric patients with single right ventricle and normal controls

**DOI:** 10.1186/1532-429X-15-S1-E92

**Published:** 2013-01-30

**Authors:** Zili D Chu, Amol Pednekar, Esben Vogelius, Prakash Masand, Shiraz A Maskatia, Shaine A Morris, Rajesh Krishnamurthy

**Affiliations:** 1 Pediatric Radiology, Texas Children's Hospital, Houston, TX, USA; 2 MR Research, Philips Healthcare, Houston, TX, USA; 3 Pediatrics (Cardiology), Texas Children's Hospital, Houston, TX, USA

## Background

Compliance is an important physical property of the aorta, reflecting its ability to work as a buffer during the systolic phase of cardiac cycle. There is a great interest in studying ventriculoarterial interations and aortic properties like stiffness and distensibility in patients with congenital heart disease due to their ability to predict cardiovascular mortality [1]. A surrogate marker of arterial stiffness is pulse wave velocity (PWV), which has been linked to prognostically adverse cardiac phenotypes in adults. There is little data on PWV in patients who have undergone single ventricle repair.

## Methods

**Figure 1:**
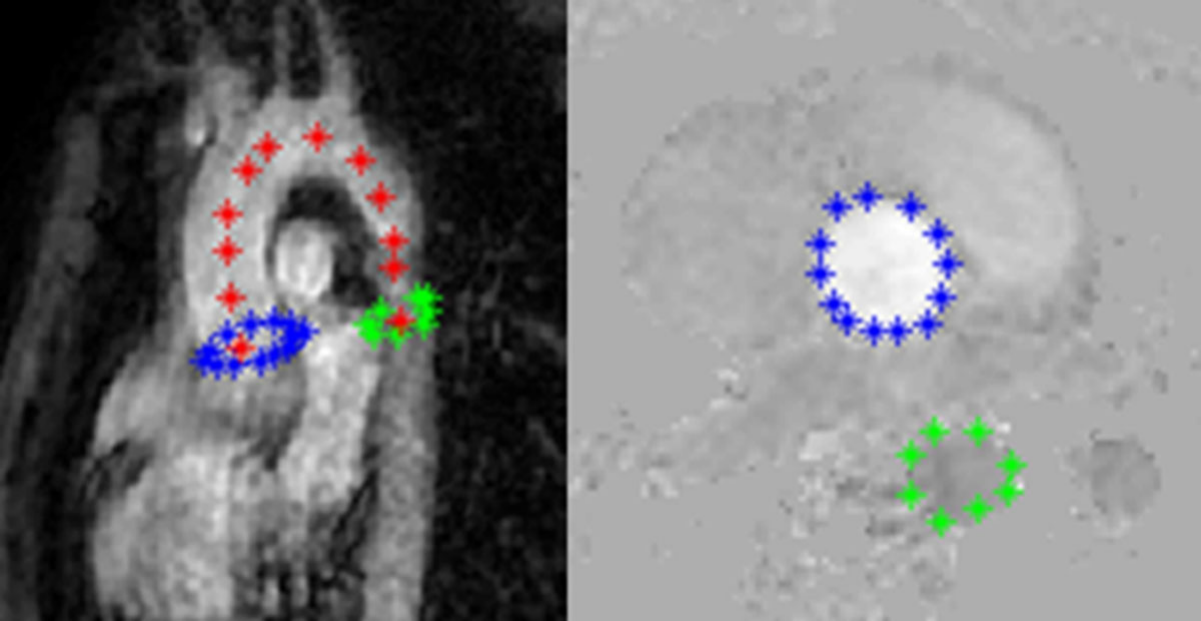
The sagittal view of whole aorta on the left and the axial view of one PC image on the right. Blue and green colors represent the ascending and the descending portion of aorta, respectively. Red points indicates the path used to compute distance between the two planes.

Following IRB approval and informed consent, five patients (mean age 9 years, and range of 7-11) with single right ventricle (SRV) status post Norwood 3 completion, and six asymptomatic healthy controls (mean age 9 years, and range of 7-11) underwent PWV assessment with MR cine phase-contrast (PC). All MR studies were performed on a 1.5T Philips Achieva scanner. Two planes were obtained, one axial plane through the ascending and descending aorta, and an oblique sagittal ‘candy-cane' plane through the aortic arch with the following parameters: TR/TE: 10/2.4 ms; flip angle: 15; thickness: 6 mm; pixel spacing: 1.16 x 1.16 mm^2^; cardiac phases: 48-60; and temporal resolution: 11-22 ms. The Maximum-Slope method and Cross-Correlation methods [2] were applied for calculation of the delay time (the time for the pulse wave to arrive at the descending aortic ROI from the ascending aortic ROI). The PWV is the ratio of path length (see Figure 1) and the delay time.

## Results

**Figure 2:**
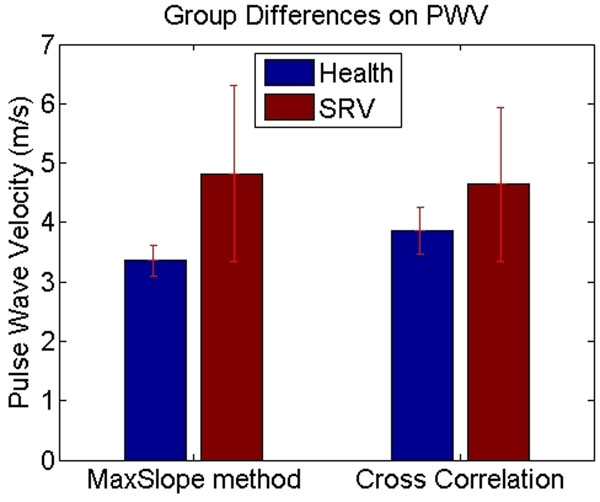
Group differences bar chart with error bars

The PWV values were as follows: 1) maximum-slope method (SRV: 4.82 ± 1.48 and healthy: 3.36±0.26, two-tailed T-test p=0.0622); and 2) cross-correlation method (SRV: 4.64 ± 1.29 and healthy: 3.86 ± 0.4, two-tailed T-test p=0.240), see Figure 2. Both the estimation methods indicate increased PWV in SRV, which is consistent with previous reports [1]. The standard deviation in the patient group is very high compared to healthy subjects, which yields a statistically insignificant result. The abnormal anatomy of the reconstructed aorta post Norwood palliation, with variable neo-aortic dilatation and location of the Stansel anastomosis is an important cause of intra-observer variability of the flow measurement, and decreases precision of PWV estimation in patients when compared to controls.

## Conclusions

Our preliminary results suggest heightened PWV in SRV. However, the two plane method to estimate PWV is sensitive to placement of the acquisition plane along the neo-aorta, and may not be a reliable indicator of aortic stiffness. An alternative method that utilizes the long axis of the aorta to calculate PWV may provide greater reproducibility.

## Funding

None.

